# The Laws of Epidemics and Preventives

**Published:** 1873

**Authors:** 


### THE LAWS OF EPIDEMICS AND PREVENTIVES.

The eminent botanist of Geneva, Alphonse de Candolle, has recently written a work entitled, *Histoire des Sciences et des Savans depuis deux Sicles*, which has a peculiar interest to physicians. His object in writing it is to display the laws of inheritance and selection in their action on the human species, and his breadth of views, profound knowledge of natural science, and charming style, render it one of the most attractive books we have got hold of in a long while.

One of the essays in it we wish particularly to bring to the notice of thoughtful medical men, namely, the sixth, which is entitled, "On the necessary alteration in the intensity of maladies, and in the value of preventives, such as vaccination."

It must have impressed every one of our readers, and even the general mind of the public, that the recent epidemics of small-pox in America and Europe are hardly to be explained by the neglect of vaccination. Indeed, very generally, the theory has been advocated that the Jennerian virus has "run out," or exhausted itself, somehow, and a general recourse was recommended to "bovine virus," a recommendation in which this journal did not sympathize nor aid, because all proof of its necessity was wanting, as we have repeatedly demonstrated.

Certainly the prevalence of small-pox was not altogether accounted for by neglect of vaccination. A better explanation is possible. Medical history shows that the first visit of an epidemic is the most severe, and that it diminishes in intensity from generation to generation. This is because the constitutions most predisposed to its fatal attacks succumb at the outset, and by a process of natural selection, those least so transmit their immunity to their children. Atavism develops occasionally, great liability to the morbid influence, and such individuals are again removed by the disease. At length, not enough predisposed persons remain in a community to furnish food for the epidemic.

Now, if a preventive measure, such as vaccination, is employed, it, combined with the result of natural selection, materially hastens this immunity. So it was with this means. When first used, Europeans had been for so many generations exposed to the poison of small-pox, that, thanks to the principle of selection, they could resist tolerably well its effects. Viccination rendered the disease, for two generations, really very rare. Many individuals who, through atavism, had inherited a fatal liability to it, were never exposed. Their children received from them this liability; until there were in the present generation very many far more sensitive to the variolous poison than in Jenner's days. On them vaccination has a less protective power than on others, and a certain recrudescence of the epidemic was sure to manifest itself.

These alternations in the fatality of epidemics and the efficacy of preventives will be more plainly visible in proportion as the disease is more fatal, and the more liable to attack children. Hence, it is seen clearly in the periodic epidemics of diptheria, which sweep over the land with intense malignity about every other generation, weeding out most of those children who, by atavism, have been born with a peculiar susceptibility to its poison.

There is no occasion, in the recognition of this law, to find cause for depreciation of vaccination as a prophylactic means. We learn its limits, and understand all the better its positive efficiency within these limits. But the lesson bids us beware of that presumption which might lead us at any time to think that in this or in any other preventive means, we have discerned an infallible and absolute method to stamp out a disease at once and forever. Any such idea is the product of a defective knowledge of the laws of the human constitution, and of the general principles of evolution as applied to organic bodies.—*Medical and Surgical Reporter.*

